# GigaFRoST: the gigabit fast readout system for tomography

**DOI:** 10.1107/S1600577517013522

**Published:** 2017-10-17

**Authors:** Rajmund Mokso, Christian M. Schlepütz, Gerd Theidel, Heiner Billich, Elmar Schmid, Tine Celcer, Gordan Mikuljan, Leonardo Sala, Federica Marone, Nick Schlumpf, Marco Stampanoni

**Affiliations:** a Swiss Light Source, Paul Scherrer Institute, Villigen, Switzerland; b Electronics for Measuring Systems, Paul Scherrer Institute, Villigen, Switzerland; c Information Technology Division, Paul Scherrer Institute, Villigen, Switzerland; d Controls Section, GFA, Paul Scherrer Institute, Villigen, Switzerland; e Institute for Biomedical Engineering, ETH Zurich, Switzerland

**Keywords:** X-ray tomographic microscopy, 4D imaging, high-speed camera, fast readout system, evolution of dynamic systems

## Abstract

Owing to recent developments in CMOS technology, it is now possible to exploit tomographic microscopy at third-generation synchrotron facilities with unprecedented speeds. Despite this rapid technical progress, one crucial limitation for the investigation of realistic dynamic systems has remained: a generally short total acquisition time at high frame rates due to the limited internal memory of available detectors. To address and solve this shortcoming, a new detection and readout system, coined GigaFRoST, has been developed based on a commercial CMOS sensor, acquiring and streaming data continuously at 7.7 GB s^−1^ directly to a dedicated backend server. This architecture allows for dynamic data pre-processing as well as data reduction, an increasingly indispensable step considering the vast amounts of data acquired in typical fast tomographic experiments at synchrotron beamlines (up to several tens of TByte per day of raw data).

## Abstract 2

The GigaFRoST detector enables high acquisition rates and long scanning times for dynamic experiments.

## 1. Introduction   

Tomographic microscopy in its simplest form, using quasi-parallel synchrotron X-rays, is perhaps the most versatile X-ray method in terms of applications. Imaging beamlines worldwide are widely used for applications in both biology and materials science. The anatomy of fixed biological tissue, insights into fossils and the internal organization of voids and various compounds in materials were subjects of study from the onset of imaging at synchrotrons. Lately, the focus has shifted towards more realistic sample and system states. Biological tissue is studied in its wet rather than fixed state. Functional anatomy and biomechanics (Walker *et al.*, 2014 ▸) have replaced simple static anatomic studies. Similarly, in materials science, investigating the mechanical deformation of internal structures or looking into geophysical processes have attracted a lot of attention during recent years (Maire *et al.*, 2016 ▸; Baker *et al.*, 2012 ▸). In general, the limits of spatio-temporal resolution and density sensitivity are improved every year, driven by the needs of new scientific questions and applications. While the spatial resolution has remained at the micrometer level due to the indirect detection scheme, there has been rapid progress in improving the temporal resolution. Around the year 2005 (Lambert *et al.*, 2007 ▸) the scan time for a single tomographic volume was of the order of 10 min at a spatial resolution of 5–10 µm. Already then, it was, however, demonstrated that it was possible to acquire a tomogram in less than a minute (Di Michiel *et al.*, 2005 ▸; Lambert *et al.*, 2010 ▸), and today 20 tomographic scans may be acquired within 1 second (Mokso *et al.*, 2013 ▸; dos Santos Rolo *et al.*, 2014 ▸; Maire *et al.*, 2016 ▸). With such an acquisition speed, a new set of challenges arose. The fast CMOS detectors able to collect images at a multi-kHz rate were designed for burst operation, meaning that the number of images in one sequence of acquisition is limited by the internal memory of the detector. Thus, no sustainable fast acquisition is possible. In turn, the dynamics of *in situ* studies can only be followed for a short time period and a complete understanding of the processes involved is hampered. In this article, we introduce a solution for fast and sustainable image recording in the form of a new data acquisition system, named GigaFRoST (**Giga**bit **F**ast **R**ead**o**ut **S**ystem for **T**omography). Its performance and uniqueness are demonstrated with two examples.

## 2. The GigaFRoST camera system   

### 2.1. Prerequisites   

The typical detection system for tomographic microscopy consists of a visible-light camera coupled to a scintillator screen through an optical lens system. The scintillator screen converts X-rays into photons with wavelengths in the visible-light regime, which are collected by optical lenses and projected with a preselected magnification onto the sensor of a visible-light camera (CMOS, CCD, sCMOS, *etc.*). CMOS technology is most commonly used for fast imaging due to the availability of small pixel sizes at high frame rates.

The general requirements for an X-ray detection system for fast tomographic microscopy may be summarized as follows:

(i) Pixel sizes smaller than 20 µm.

(ii) Multi-kHz frame rates with a minimum sensor size of 2 Mpixels.

(iii) Versatile triggering modes.

(iv) Sustainable acquisition over a time period of at least 10^5^ times the frame rate (yielding time sequences of hundreds of individual tomograms).

(v) Live streaming of at least a subset of the data to allow for near real-time monitoring of the dynamic processes being imaged.

(vi) It should be possible to write only a subset of the acquired data to disk, allowing for a dynamic pre-selection of only the useful data and reducing the overall data volume.

(vii) Easy communication and scripting.

The first two requirements listed above, and to some extent also the third, are matched by the commercially available pco.Dimax (PCO AG, Germany; https://www.pco.de) fast CMOS detector, which since its introduction in 2008 has enabled many of the spectacular achievements in fast synchrotron-based imaging (Baker *et al.*, 2012 ▸; Rack *et al.*, 2013 ▸; dos Santos Rolo *et al.*, 2014 ▸; Mokso *et al.*, 2015 ▸; Finegan *et al.*, 2015 ▸; Maire *et al.*, 2016 ▸). The use of the pco.Dimax for the investigation of a large number of more complex dynamic phenomena has, however, been hindered so far by two main technical limitations. Firstly, the detector cannot continuously stream data from the camera and thus no live preview is available to monitor the system in real time. Secondly, the on-board memory is too small to acquire a sufficiently large number of frames to follow many processes in their entirety.

### 2.2. GigaFRoST design principles   

The design strategy for the GigaFRoST detector was to build upon the excellent high-speed characteristics of the pco.Dimax imaging chip and sensor headboard, and to provide the capacity for continuous real-time readout through a custom-built readout system. The following sections describe the details of the hardware and software developments that have been necessary to manage the data flow from the imaging chip and which make up the complete GigaFRoST system.

### 2.3. Hardware configuration   

The pco.Dimax headboard consists of the imaging chip with 2016 × 2016 pixels, 12 analog-to-digital converters (ADCs), and some auxiliary electronics. The imaging sensor is sub­divided into four quadrants, which are each read out in parallel by three of the ADCs. The readout for every quadrant starts at the center of the sensor and ends at the outer corner. Data are read out horizontally in lines, progressing towards the upper or lower edges of the sensor. Readout of only a subset of all pixels is possible, but due to the readout process starting at the center the resulting region-of-interest (ROI) has to be centered on the imaging chip. Onboard binning is not supported.

**Figure 1:**
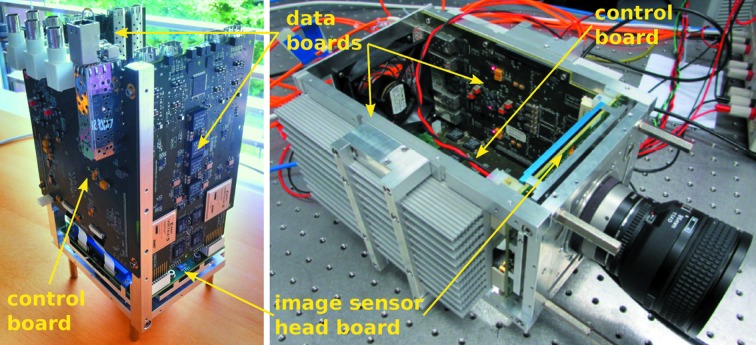
Photographs of the GigaFRoST camera’s custom-made data and control boards attached to the image sensor head. All covers of the housing are removed.

**Figure 2:**
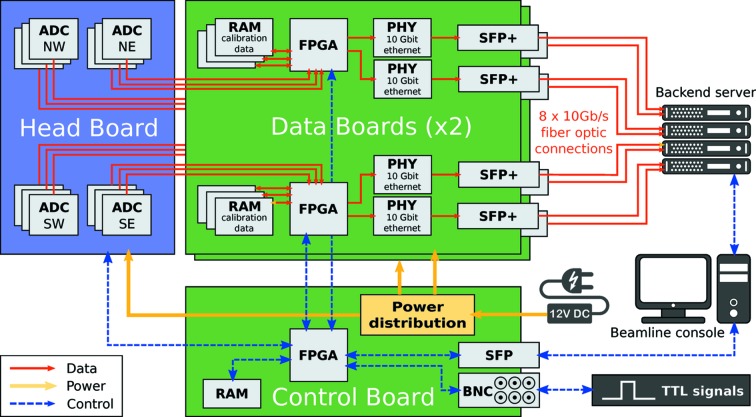
Sketch of the GigaFRoST camera architecture. The pco.Dimax headboard (blue) is connected to two custom-built data boards and a control board (green). Data connections for the image stream are shown in red, control connections in blue.

Fig. 1 ▸ shows photographs and Fig. 2 ▸ shows a schematic of the custom-built readout electronics for the imaging chip. Two data boards, each of which is equipped with two field-programmable gate arrays (FPGAs), are connected to the pco.Dimax headboard. Data from the ADCs of each quadrant of the imaging sensor are streamed to one of these dedicated FPGAs. Linearity and offset corrections for all pixels are applied in real time by the FPGAs, based on calibration coefficients that are stored in on-board RAM directly connected to the FPGAs. The data from each FPGA are then split into two parallel streams (even- and odd-numbered pixel rows) which are sent directly to the backend server using the UDP protocol *via* eight on-board ethernet modules connected to single-mode fiber-optic cables rated for 10 Gb s^−1^ transfer. Their combined transfer rate reaches 7.65 GB s^−1^.

An additional control board coordinates the readout system. It features one FPGA with a built-in PowerPC (PPC) processor and attached RAM. A specialized embedded Linux system running on the PPC CPU serves as the control center for the GigaFRoST camera. The FPGA directly controls the acquisition signals for the image sensor and headboard and contains all the logic to coordinate and process the different input and output signals. A softIOC running on the embedded Linux system provides a full EPICS (EPICS, 2017 ▸) interface to the camera functions, and all standard communications during experiments take place *via* channel access calls (EPICS PVs). The control board is equipped with a single 1 Gb s^−1^ network module for standard ethernet connectivity. An additional serial connection can be used for debugging using a serial console connection to the operating system.

Six BNC connectors allow for the external synchronization of the image acquisition through TTL signal logic. Two inputs are provided for external enable and trigger signals, while four outputs report the camera’s busy state, acquisition signal, gated acquisition signal (by the enable signal) and a sync-out of the exposure trigger signal with an optional delay.

### 2.4. IT infrastructure   

Managing the large stream of incoming image data poses a considerable challenge for the information technology (IT) infrastructure and requires a careful layout of the network architecture along with a tight coordination and integration between the individual data handling processes. The strategy described in the following paragraphs for the case of the GigaFRoST is, in fact, not unique to this camera, but has been adopted for all high-performance detectors operated at Paul Scherrer Institute (PSI) in a similar fashion. In designing the data management pipeline, great care has been taken to ensure that the system remains scalable and modular, allowing for straightforward parallelization of performance-critical operations and for easy serialization of essentially independent processing steps.

The primary actors in the case of the GigaFRoST currently include the data backend server, which receives the camera data and assembles the complete camera frames to be streamed out again, a file writer service running on a separate server, as well as the attached high-performance file storage system.

**Figure 3:**
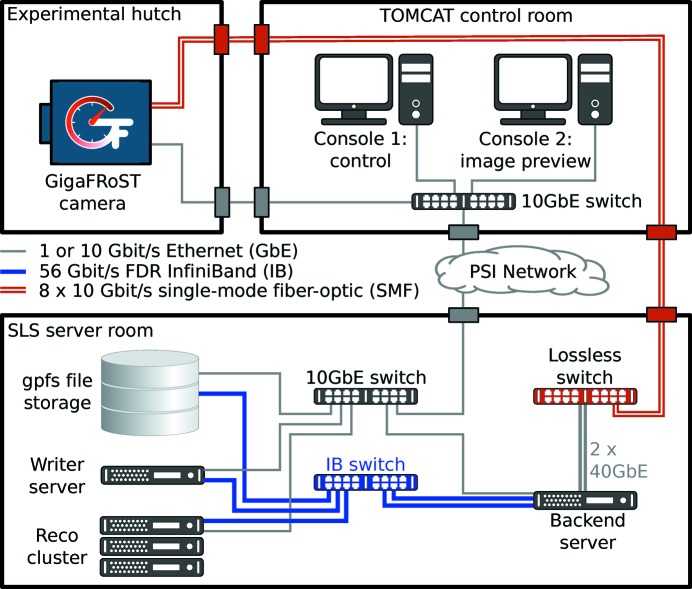
Network topology for the GigaFRoST data acquisition system.

The network topology and locations of the different machines and devices is shown in Fig. 3 ▸. Data streamed by the GigaFRoST data boards from the experimental hutch *via* the eight parallel 10 Gb s^−1^ single-mode fiber-optic links are received by a specialized network switch (Mellanox SX1036) in the Swiss Light Source server room. The switch features a non-blocking lossless data transport even at high data rates and routes the stream to the GigaFRoST backend server *via* two 40 Gb s^−1^ ethernet links. The backend server is attached to the network backbone of the server room infrastructure with two 56 Gb s^−1^ InifiniBand FDR connections. The file storage system, file writer server and tomographic reconstruction cluster are attached to the same InfiniBand switch.

There are two major communication channels for the IT infrastructure. On the one hand, the actual image and header data from the GigaFRoST camera need to be passed between the different processes (processes in this context refer to the individual software tasks running on the hardware, such as the receiver processes, the file writer process, *etc.*, described below). Data flow is characterized by its large volume and high rate, and is typically uni-directional. These data are streamed using the ZeroMQ distributed messaging protocol (ZeroMQ, 2017 ▸). One sender can stream data to multiple receivers at once.

On the other hand, the individual processes need to be controlled by the central data acquisition engine and communicate amongst themselves. These are short messages and commands with typically little associated data, but information flow is bi-directional and somewhat asynchronous with respect to the main data flow. Messages are passed *via* a so-called *representational state transfer* (REST) interface (Fielding & Taylor, 2000 ▸), which essentially uses standard HTTP methods (*i.e.*, HTTP GET, PUT, POST, *etc*.) to post messages and request or send data.

#### 2.4.1. Backend server   

The data backend is running on a dedicated server, which is equipped with two CPUs with 14 cores each. Per CPU, one dedicated 40 Gb network card and 128 Gb of RAM are available. One CPU receives all of the data from the north half of the detector, while the other one processes the south half. Each of the four incoming data streams per CPU are assigned to a separate receiver process running on a dedicated CPU core. These receiver processes collect the partial image data and write it into the correct memory locations. In this way, each of the two half-frame images (north and south) are assembled in-place in the CPU memory by four completely independent receiver processes.

Data management in the backend server memory is governed by a specialized ring buffer architecture used for high-frequency memory access and data transfer applications, based on the design proposed by and found in the LMAX disruptor (Thomson *et al.*, 2011 ▸; Disruptor, 2017 ▸). Incoming image frames are stored into consecutive slots of the data ring buffer. The maximum number of available ring buffer slots depends on the ROI size, and determines how many images the data backend can store at any one time. Table 1 ▸ lists the total number of frames that fit into the currently available 256 GB of total memory for different frame sizes. For very small ROIs, the overhead due to the image header data, which remains constant regardless of the frame size, increases. Also listed are the maximum achievable frame rates for the given ROI size (the limit is given by the readout of the imaging chip, not the streaming capacity of the readout system).

Once a frame has been fully assembled in the ring buffer by the backend receiver processes, it is ready to be sent to any downstream actors (storage to file, reconstruction, *etc*.). A separate publishing process, running on one of the two CPUs, posts the frame data into the ZeroMQ stream.

A second much smaller ring buffer can hold selected frames to be streamed to a live preview application (see §3.1.2). However, processing those frames in the large data ring buffer always has priority over the handling of the preview buffer to avoid or at least minimize data loss. Preview may therefore be ‘lossy’, meaning that frames can be dropped or delayed if the system is operating under full load.

In addition to these two frame ring buffers, there are a number of much smaller ring buffers to hold metadata and to keep track of the individual processes working on each image frame.

#### 2.4.2. File writer and storage   

The file writer process, in charge of writing the image data to disk, runs on a separate dedicated server. The writer process receives the image and header information by subscribing to the ZeroMQ stream published by the backend and then packs everything into HDF5 files. The file structure and layout of the HDF5 files is completely configurable at the writer level. For the GigaFRoST data, we use the Scientific Data Exchange format (De Carlo *et al.*, 2014 ▸). The central file storage server uses the General Parallel File System (GPFS) (Schmuck & Haskin, 2002 ▸) developed by IBM (which has meanwhile been re-branded to ‘IBM Spectrum Scale’). In addition to the InfiniBand connections to the servers in the server room, the file storage is also accessible by beamline or analysis consoles over standard ethernet connections (1/10 GbE).

#### 2.4.3. Reconstruction cluster   

The reconstruction pipeline, running on another dedicated compute cluster, usually processes data from files already written to disk storage. However, it is also capable of subscribing directly to the ZeroMQ stream from the backend and running the reconstruction job without the need of intermediate file access. Direct streaming of data to the reconstruction pipeline is still in beta-phase, but first tests confirm the feasibility of this approach, at least for a subset of the streamed data, depending on the scan frequencies (Marone *et al.*, 2017 ▸).

### 2.5. System performance and expandability   

The data backend is capable of receiving data and storing it into the ring buffer at the full streaming rate of 7.7 GB s^−1^. However, despite using dedicated hardware and carefully tuned software, the GigaFRoST system does not yet manage to redistribute these data from the ring buffer to any downstream consumers at the same rate. For example, the maximum throughput when writing data to file is approximately 2–3 Gb s^−1^ at present. As a consequence, the ring buffer saturates at some point, thus limiting the total number of frames that can be recorded at high rates without dropping any (however, the minimum amount of data that can be collected continuously is at least the size of the internal server memory of currently 256 GB). Bottlenecks in the data processing and transfer steps are currently investigated to improve the system performance. The design goal is to achieve file writing speeds that match the acquisition rates, such that continuous data collection is ultimately only limited by the available disk storage.

Given the carefully modularized design of the crucial components and processes, expanding the GigaFRoST system to add new features or improved capabilities is fairly straightforward. For example, the data backend could be distributed and parallelized over multiple servers, each one processing only a subset of the full frame. Similarly, the file writing could be split into more than one process, each one either writing partial frames in parallel or a given number of full frames at certain intervals (round robin schemes). The streaming architecture also allows the insertion of new modules into the data stream. For instance, a dedicated data compression module could subscribe to the data stream from the backend and publish a new compressed data stream to be received by the file writer.

An upgrade of the backend server is planned for October 2017 to expand the available server memory to 512 GB, thus approximately doubling the number of frames that fit into the ring buffer.

## 3. GigaFRoST operation   

### 3.1. Data acquisition modes   

One crucial feature request for the design of the GigaFRoST camera was the capability to stream a nearly real-time live feed of the acquired frames to the user for preview purposes. At the same time, it was requested that only subsets of the data can be saved to disk or selected for data processing. Taken together, these two requirements imply the necessity for a novel mode of operation where the camera hardware itself is acquiring frames independently of whether they are to be previewed or saved. This decision is delegated to the backend and can be interactively or automatically controlled *via* user input or programmatic feedback.

#### 3.1.1. Selecting which frames to store   

The GigaFRoST camera can be run in two distinct operation modes, which differ in the way the camera enable signal is used. We will refer to these operation modes as the two *enable schemes*.

The first enable scheme corresponds to the conventional way of using an enable signal for cameras or other counting devices, which controls the response of the camera hardware to acquisition requests. In essence, the camera triggers and acquires frames only if the enable signal is ON; otherwise, the acquisition requests are simply ignored and any of the associated electronic processes on the camera chip and readout boards are suppressed. We will refer to this mode of operation as the *physical enable scheme*.

In contrast, when using the second novel mode of operation, coined the *virtual enable scheme*, the camera hardware completely ignores the enable signal. Once the camera is armed, exposures are taken for every exposure trigger signal, and the GigaFRoST sends the frame data to the backend server, regardless of the enable signal. However, the state of the enable signal at the start of every exposure is documented in the image header metadata, where one designated status bit contains the *do-not-write* flag. This flag is set to 1 when the enable signal was OFF, and to 0 if the enable signal was ON. Thus, each image frame that arrives in the backend carries the information about the enable state at its acquisition time.

The response to the do-not-write flag is now up to the backend processes, and can be controlled at the software level. In its current implementation, the backend will neither append those images to the ring buffer nor send them to the HDF5 writer. But the images are still considered for preview if they match the criteria set by the preview strategy (see §3.1.2).

**Figure 4:**
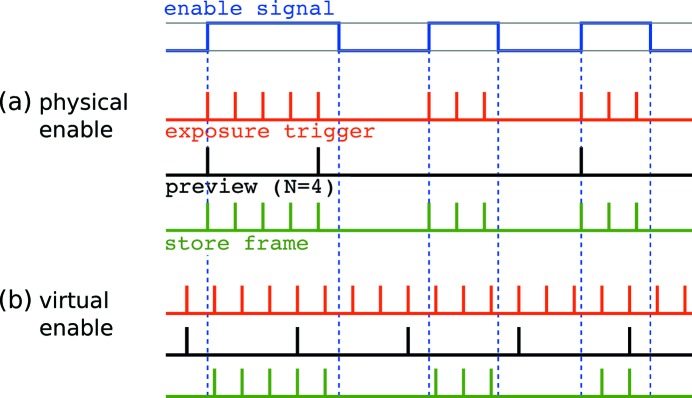
An example showing the differences between signal trains for the (*a*) physical and (*b*) virtual enable scheme operation. The external enable signal (blue) is identical for the two modes of operation. The camera triggers (red) are provided by the internal auto trigger mode (see section on trigger modes[Sec sec3.2.2]). Only frames with an active frame store bit (green) will be added to the data ring buffer. Note the shift in frame timing between the *physical* and the *virtual enable* scheme operation. Even though the second and third gate have the same length, the number of accepted trigger pulses is different (three and two pulses, respectively) in the *virtual enable* scheme due to the timing jitter of the gating signal with respect to the fixed frequency trigger signal. Also shown are the frames selected for preview (black), assuming a preview strategy displaying every fourth frame acquired by the camera.

By using the virtual enable scheme, the requirement to decouple the preview functionality from the used data stream later can be fulfilled. The physical enable scheme, on the other hand, reproduces a conventional mode of operation. Fig. 4 ▸ illustrates the differences between two acquisition modes and their consequences for the frame timing.

#### 3.1.2. Live preview   

All images selected for preview (regardless of whether they are also selected for storage in the data ring buffer) are sent to a separate small internal ring buffer which is used for preview only. This separates the preview and writer data flow already at the backend level. A dedicated ZeroMQ stream publisher process for the preview data serves the preview frames to any downstream visualization or analysis clients.

Four preview strategies are currently available:

(i) Timestep: select images for preview based on a given time interval, for example one image every 200 ms (5 Hz).

(ii) Modulo: select every *N*th image.

(iii) All: select all images for preview.

(iv) None: disable image preview.

The availability and configurability of these different preview strategies is a crucial feature for time-resolved fast experiments. For instance, one would usually like to display the same projection orientation in the preview of a sample which is continuously rotating during the data acquisition to accurately monitor and visually assess changes. One therefore needs to precisely synchronize the preview frequency to the rotation speed.

For preview by human eyes, an update rate of several Hertz is usually completely sufficient, and the additional processing load on the system is minimal. However, one might envisage processes performing on-line monitoring of the data with respect to certain image metrics, triggering new data acquisitions upon detecting certain changes in the streamed images. In such cases, the preview rates might be much higher, putting more stringent requirements on the handling of the preview buffer.

#### 3.1.3. Acquiring a fixed number of frames   

Both the virtual and the physical enable schemes make use of the enable signal to determine when frames are to be stored or acquired. The conventional usage is to accept only those images when the enable signal is ON.

However, the GigaFRoST provides alternative acquisition modes where a change in the enable signal (*i.e.* the leading and trailing edges of the enable gate) can be used to initiate the acquisition of predefined series of exposures, specified by a given fixed number of frames to be taken. Once the acquisition has started, the enable state becomes irrelevant for the remainder of the sequence.

The three user-configurable parameters used to control this mode are the fixed number *N* of frames for the sequence and two control flags indicating whether the leading and falling edges are actively triggering a sequence, respectively. Four different combinations of the control flags are possible, and they result in the following behavior:

(i) No trigger edge enabled (OFF/OFF): the enable signal is used in the conventional way to gate the acquisition.

(ii) Only leading edge enabled (ON/OFF): the sequence of *N* frames is initiated as soon as the enable signal turns ON.

(iii) Only trailing edge enabled (OFF/ON): the sequence of *N* frames is initiated on the falling edge of the enable signal. Nothing happens while the enable signal is ON.

(iv) Both edges enabled (ON/ON): acquisition is started when the enable signal is turned ON and continues up to the falling edge, regardless of the number of frames acquired during the gate. After the enable signal turns OFF, another *N* frames are taken. The total number of images taken in this mode is therefore given by a combination of the enable gate duration (in conjunction with the number of actual exposure trigger signals received during this time) and the number of frames *N* to be acquired at the end of the gate.

**Figure 5:**
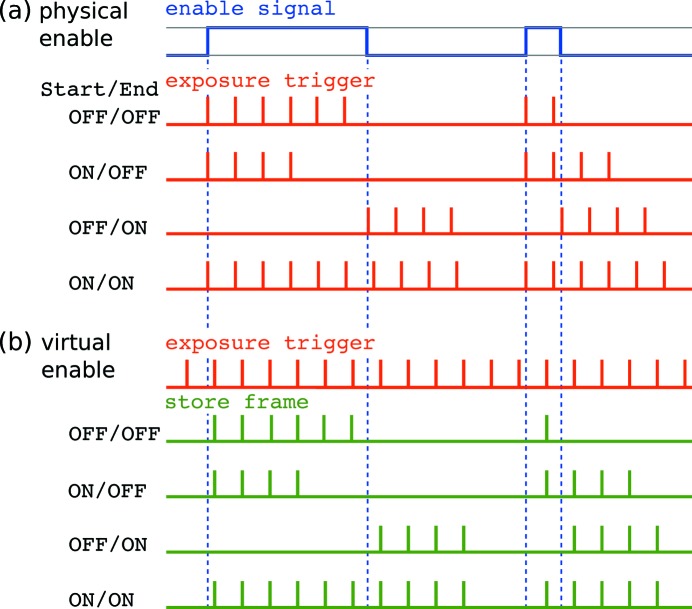
Illustration of the four possible different modes using the start or end of the enable signal to trigger the acquisition of predefined frame series, both for the (*a*) physical enable and (*b*) virtual enable schemes. The fixed number of frames to be taken has been set to *N* = 4. Note that the frame store signal is not shown in (*a*) since it is identical to the exposure trigger signal. The exposure trigger in this example is assumed to be produced internally by the camera at constant frequency (auto trigger). Due to the different timing of the trigger pulses between the physical and virtual enable schemes, the exact frame timing is different for both cases.

These trigger modes work both in virtual and physical enable scheme, although the signal train timing might differ between the two schemes. Fig. 5 ▸ illustrates the frame sequences for the four modes.

### 3.2. Triggering and synchronization   

A tight synchronization between the controlled stimulation or prompt detection of the fast dynamics in the sample under investigation and the data acquisition is indispensable to capture dynamic processes in the desired state with time-resolved imaging. Changes can be either induced externally, as is the case when ventilating a small animal or by heating a sample above a certain temperature, or occur spontaneously and autonomously, for example when monitoring the heart beat of a small animal or during the sudden formation of a fatigue crack in a material under load. Additionally, to obtain reliable three-dimensional structural information, the sample rotation must accurately match the system dynamics.

The details of the triggering and synchronization between the various components and devices can change significantly for different experiments. Typically, one process will drive the triggering of the data acquisition. In some cases the timing is determined by the sample, while under other circumstances the camera or rotation stage need to be the master process.

To accommodate these different needs, the TOMCAT beamline provides a very flexible signal processing infrastructure to distribute and record the synchronization signals from various components. The fast air-bearing Aerotech rotation stage features custom triggering and enable signal generation options of its own (Lovrić *et al.*, 2016 ▸). Consequently, the GigaFRoST has been designed for a similar flexibility, providing several options to control the timing of the frame acquisitions, as described in §3.1.

The maximum achievable frame rate for the GigaFRoST depends on a few factors. Firstly, the time per frame cannot be shorter than the requested exposure time plus an additional system overhead, 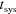, which is required to control the imaging sensor and is of the order of 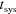 = 3.5–4 µs. For very short exposure times, the sensor readout time becomes the limiting factor, which in turn depends on the chosen ROI size and shape. Table 1 ▸ lists the maximum achievable frame rates for different ROI settings, assuming exposure times that are shorter than the corresponding acquisition periods by at least 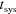.

In the following, the details of the various available triggering and enable modes are given.

#### 3.2.1. Enable modes   

Three different mechanisms are available to control the camera’s enable status. We will call these *enable modes*, in contrast to the *enable schemes* discussed above (see §3.1.1). The enable modes are:

(i) External enable: the enable state is controlled through an external TTL signal.

(ii) Soft enable: a software signal sets the enable state.

(iii) Always enable: the camera is constantly enabled as soon as it is started (armed).

#### 3.2.2. Trigger modes   

The trigger signal to the camera’s imaging chip controls when the acquisition of a single frame is initiated:

(i) External trigger: the trigger signal for each acquisition is supplied by the leading edge of an external TTL signal.

(ii) Soft trigger: each acquisition is triggered by a software signal.

(iii) Timer trigger: the image acquisition is triggered internally by the camera control. The trigger frequency is determined by the requested exposure period.

(iv) Auto trigger: the trigger signals are generated internally at the maximum frequency achievable for the selected exposure time.

#### 3.2.3. Exposure modes   

While the trigger mode above controls how an exposure is initiated, the exposure mode determines when an exposure is ended. The following modes are available:

(i) External exposure: the length of the camera exposure is controlled through the length (falling edge) of the TTL gate on the external trigger signal. Sometimes this mode is also referred to as *bulb mode*.

(ii) Soft exposure: the acquisition is terminated by a software signal. In practice, this mode is not very useful since the exposure time cannot be accurately controlled due to latencies in the software communication.

(iii) Timer exposure: the exposure duration is given by the configurable exposure time and controlled by the internal clock signal of the camera. This is the standard exposure mode for a vast majority of experiments.

The valid range of exposure times for the GigaFRoST is 2 µs to 40 ms.

### 3.3. Image acquisition and system performance   

**Figure 6:**
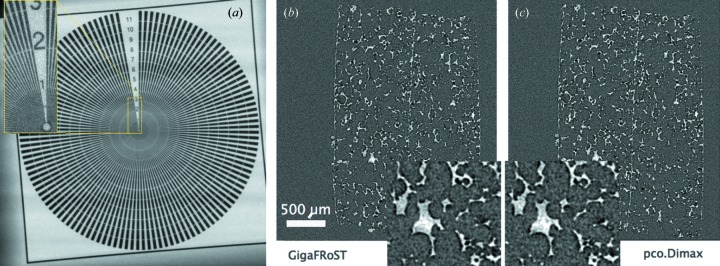
Panel (*a*) shows a radiographic projection of a gold Siemens star using a 20× objective coupled to a 20 µm LuAG:Ce scintillator. The numbers indicate the thickness of individual gold lines at the given location. In panels (*b*) and (*c*) we compare two tomographic reconstructions of a polymer foam acquired with the GigaFRoST and pco.Dimax, respectively. Acquisition parameters were: pixel size = 3 µm, exposure time per projection = 0.5 ms, number of projections = 500, size of the reconstructed volume = 1008 × 1008 pixels, X-ray energy = 20 keV.

We used two static samples to evaluate the image quality. For the two-dimensional case we acquired a radiographic projection of a gold Siemens star with a configuration consisting of a 20 µm thin Ce:LuAG crystal scintillator coupled to the GigaFRoST detector by a lens with 20× magnification. The resulting effective pixel size in the image is 0.55 µm. As Fig. 6(*a*) ▸ shows, the image is accurate, and lines down to 1 µm in size are resolved for this 1.5 µm-thick gold structure which attenuates 20% of the X-rays at 20 keV.

Systematic flaws in the image quality such as non-linearity or distortion become pronounced by combining many tomographic projections into one tomographic volume. We therefore investigated the quality of tomographic reconstruction from projections acquired with the GigaFRoST and pco.Dimax detectors. They share the same sensor and differ in the electronics and the calibration. Fig. 6 ▸ depicts one tomographic slice of a polymer foam on which we compared the noise characteristics of the two systems. Both tomographic datasets were acquired under the same experimental conditions and reconstructed with the same Fourier implementation of the Radon transform (Marone & Stampanoni, 2012 ▸). We calculated the contrast-to-noise ratio (CNR) defined as 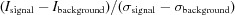 (Mokso *et al.*, 2013 ▸). We obtained almost identical values in both cases, *i.e.* CNR_GigaFRoST_ = 3.6 and CNR_pco.Dimax_ = 3.7.

## 4. Scientific applications   

Dynamic X-ray tomography is often used to experimentally validate existing models describing the behavior of the studied system. However, the time scales of changes are often not known before the experiment. Therefore, a good control of a fast tomographic experiment often relies on the availability of preview images during acquisition. In this regard, there are three main features of the new detector that distinguish it from previous systems: (i) The preview is generated continuously and independently of whether the frames are to be stored or not. Currently the preview is available for single radiographic projections, but the nearly real-time reconstruction of single tomographic slices is an ongoing development (Marone *et al.*, 2017 ▸). (ii) The preview is *time synchronous* and adjustable by the user. Thus it is possible to assure that always the same view of the sample (in terms of the projection angle) is displayed during rotation to better observe subtle changes. (iii) The detector features the necessary functionality which allows one to flag selected frames dynamically for storage or not, based on a near real-time analysis of the data stream. This feature has not been implemented in practice since it still requires substantial work on the analysis methods, but everything on the detector-side is ready for this mode of operation.

In the following two case studies on foams, we demonstrate the utility and necessity of these features for the *in situ* three-dimensional monitoring of complex cellular systems.

**Figure 7:**
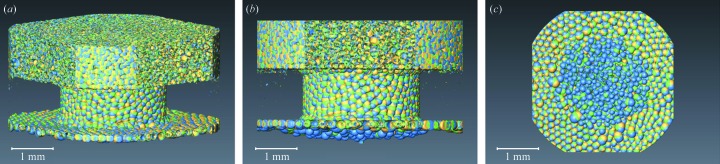
Volume rendering of a liquid foam flowing through a constriction. The acquisition rate was set to five tomographic scans per second to resolve the flow in time and track the individual cells for a period of 27 s. In the volume rendering, each color represents a time frame of the flowing foam: *t*_0_ (yellow), *t*_0_ + 2 s (green), *t*_0_ + 4 s (blue). In (*a*) the overview image of the constriction is shown, in (*b*) the side view shows how individual bubbles progress in the vertical direction from the top towards the bottom of the flow cell (constriction) and in (*c*) the view from the bottom demonstrates the radial displacement of the bubbles towards the walls below the constriction. See also movies 1 and 2 of the supporting information.

Foams are biphasic systems categorized as either solid or liquid foams. Liquid foams represent in most cases a dynamically evolving system with often spectacular properties. Probing these dynamic systems in 3D poses numerous challenges. In particular, their dynamic and multi-scale character solicits probing a large field of view with high spatial and temporal resolution. X-ray tomographic microscopy already helped to reveal part of the physics (Lambert *et al.*, 2010 ▸) which could be earlier addressed only on reduced dimensions (2D) (Dollet & Graner, 2007 ▸). Recently, liquid foam rheology in 3D was better understood (Raufaste *et al.*, 2015 ▸), but in the same study it was pointed out that the statistics and hence the confidence in the results were significantly affected by the severe limitations on the time span of one sequence acquisition. In the following example using the GigaFRoST detector, we could for the first time capture a foam rheology study with high spatio-temporal resolution and at the same time a sufficiently long total acquisition of the dynamic series. Fig. 7 ▸ represents three snapshots in time of a liquid foam flowing through a constriction. The volume rendering is selected from the 130 tomographic scans in the series acquired at a rate of five tomographic scans per second. Each scan consists of 300 radiographic projections of 2016 × 1800 pixels (H × V). 39000 projections form the entire tomographic scan sequence that allows a time span of 27 s to be captured. The mean foam velocity at the broadest point of the flow chamber was 5–10 µm s^−1^ and faster in the constriction and nearby. With the average bubble size being 150 µm, the foam progresses about two bubble diameters during the total acquisition even at the places with the slowest flow. This can be appreciated from the radiographic projection images shown in movie 1 of the supporting information. This movie represents the frames that are typically visualized in preview during acquisition allowing the motion of the foam to be monitored. Detailed quantitative analysis of the reconstructed volumetric data is beyond the scope of the current manuscript; however, already from the simple isosurface representation, shown in Fig. 7 ▸ (made using *Avizo* software), certain observations can be made, such as the vertical motion of the bubbles or their redistribution towards the walls of the flow chamber below the constriction.

**Figure 8:**
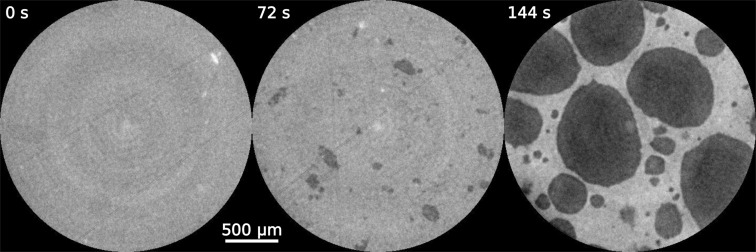
Three tomographic cross sections of an AlSi_8_Mg_4_ + 0.5 wt% TiH_2_ foam captured *in situ* during the foaming process. The images are tomographic cross sections. The entire foaming process is recorded at 20 Hz. The time step between the selected snapshots is 72 s, while the full four-dimensional volume represents the state of the system every 50 ms.

The second example to demonstrate the new capabilities of the GigaFRoST is the nucleation process of an aluminium foam. Solid foams are technologically important systems, yet their manufacturing is poorly understood due to a lack of *in situ* monitoring methods to observe the structural evolution during foaming. Pioneering studies used high-frame-rate radiography to describe in a qualitative manner film rupture (García-Moreno *et al.*, 2008 ▸) or injection foaming (Babcsan *et al.*, 2012 ▸). Adding a third spatial dimension to the time-resolved studies is motivated by the possibility to extract quantitative information and gain a complete understanding of the foaming process. Using the GigaFRoST detector, we captured the foaming of an AlSi_8_Mg_4_ + 0.5 wt% TiH_2_ foam during 3 min with 50 ms temporal resolution in 3D at 5 µm pixel size (Kamm *et al.*, 2016 ▸). Such a protocol allowed us to, for the first time, visualize the foaming in three dimensions from the moment of nucleation until the solidification of the aluminium foam. Tomographic slices at three selected time instances are illustrated in Fig. 8 ▸. The initial state of the liquid aluminium is shown in the left-hand panel, the middle panel is a snapshot 72 s later at a late stage of nucleation, which then leads to the solid foam structure in the right-hand panel.

## 5. Conclusions and perspectives   

We developed a high-speed camera readout system which enables the recording of long time sequences. Image frames are streamed through fiber-optic network connections at a rate of up to 7.7 GB s^−1^ directly to a backend server, from where they can be processed in nearly real-time or stored to disk. Having immediate access to the raw data stream opens unique opportunities for the implementation of data reduction schemes and smart acquisition control. Both of these features are becoming increasingly important for a sustainable operation of imaging instruments that are today able to capture fast dynamic phenomena, such as demonstrated in this paper by the example of complex cellular systems.

## Supplementary Material

Movie 1: Time series of radiographic projections of a liquid foam moving though a constriction. . DOI: 10.1107/S1600577517013522/pp5108sup1.avi

Movie 2: Isosurface representation of full three-dimensional volume data sets at three time points of a liquid foam moving through a constriction. . DOI: 10.1107/S1600577517013522/pp5108sup2.avi

## Figures and Tables

**Table 1:** Maximum number of frames that fit into the backend ring buffer slots and maximum achievable frame rate as a function of the ROI size

ROI size (h × v)	Ring buffer slots	Maximum frame rate (Hz)
2016 × 2016 (full frame)	35930	1255
2016 × 1008 (half frame)	71860	2490
1920 × 1080 (full HD)	70422	2424
1008 × 1008	143720	4305
672 × 540	402416	10288
480 × 288	1056342	21907
240 × 240	1310720	33875
